# First experimental observations on melting and chemical modification of volcanic ash during lightning interaction

**DOI:** 10.1038/s41598-018-19608-3

**Published:** 2018-01-23

**Authors:** S. P. Mueller, C. Helo, F. Keller, J. Taddeucci, J. M. Castro

**Affiliations:** 1 Intitute of Geosciences, University of Mainz, J.-J.-Becherweg 21, D-55122 Mainz, Germany; 2 Istituto Nazionale di Geofisica e Vulcanologia, Via di Vigna Murata 605, 00143 Rome, Italy

## Abstract

Electrification in volcanic ash plumes often leads to syn-eruptive lightning discharges. High temperatures in and around lightning plasma channels have the potential to chemically alter, re-melt, and possibly volatilize ash fragments in the eruption cloud. In this study, we experimentally simulate temperature conditions of volcanic lightning in the laboratory, and systematically investigate the effects of rapid melting on the morphology and chemical composition of ash. Samples of different size and composition are ejected towards an artificially generated electrical arc. Post-experiment ash morphologies include fully melted spheres, partially melted particles, agglomerates, and vesiculated particles. High-speed imaging reveals various processes occurring during the short lightning-ash interactions, such as particle melting and rounding, foaming, and explosive particle fragmentation. Chemical analyses of the flash-melted particles reveal considerable bulk loss of Cl, S, P and Na through thermal vaporization. Element distribution patterns suggest convection as a key process of element transport from the interior of the melt droplet to rim where volatiles are lost. Modeling the degree of sodium loss delivers maximum melt temperatures between 3290 and 3490 K. Our results imply that natural lighting strikes may be an important agent of syn-eruptive morphological and chemical processing of volcanic ash.

## Introduction

Lightning discharges in ash plumes are common and spectacular phenomena accompanying explosive volcanic eruptions. Lightning events are relatively easy to detect from great distances, e.g., by satellites, and in many cases represent the only means of detecting and monitoring volcanic activity in remote locations on earth and, potentially, other planets^1–5^. Volcanic lightning also represents an ancillary hazard in and of itself, having claimed two lives in recent times^6^.

During the formation of ash plumes above an active vent or pyroclastic density current^7^, the dynamical interaction of ash particles creates charges which can, given a sufficiently large charge gradient, cause lightning within the plume. Occurrence of volcanic lightning has been reported in association with 212 historic eruptions, but, due to inconsistent observation and reporting procedures, the true number is probably much higher than that^8^. Parameters such as the frequency, intensity, polarity and spatial distribution of volcanic lightning have the potential to provide important information on the nature and development of ash plumes, such as plume height, eruption explosivity, and ash particle concentration^8–10^. Lightning processes are thought to modify the plume itself, by promoting aggregation of ash particles, affecting particle dispersion and segregation from the ash plume, processes all of which could affect the accuracy of plume dispersion models^1,8,11^.

Cimarelli and co-authors^12^ experimentally generated volcanic lightning by injecting gas-particle mixtures into a collection chamber. They systematically varied experimental conditions and established relations between the occurrence of electrical discharges (frequency, electric potential) and injected particle characteristics (size distribution, shape). While these experiments form a framework to relate fundamental eruption parameters (e.g., mass eruption, ash size distribution) driving natural volcanic plumes to lightning discharge characteristics, Cimarelli *et al*. did not report any chemical or physical alteration (e.g., melting) of the ash particles.

However, given the extreme heat release during the short duration of a natural discharge (~12,000 to 28,000 K in the central part of the plasma channel^13^), it seems likely that the ash particles suspended in a plume are affected by volcanic lightning. Genareau and co-authors^14^ found evidence of glass spherules and glass aggregates in ash deposits of two explosive eruptions (Eyjafjallajökull 2010, Mt. Redoubt 2009), and linked them to short-term melting processes induced by volcanic lightning. In a more recent experimental study, Genareau *et al*.^15^ investigated the impact of discharge characteristics and distance from plasma channel axis on stationary pseudo-ash particles.

The aim of this study is to bridge the gap between laboratory experiments and naturally occurring lightning-modified ash by exposing volcanic ash-gas mixtures to high-intensity lightning analogues. We use an experimental setup comprising an arc-welding machine as current source to melt ash and form glassy particles from both suspended and stationary ash inputs. Ash particle morphologies produced in this manner show a range of degrees of melting caused by the arc, and importantly mimic the natural ash morphologies described by Genareau *et al*.^14^. We also capture, for the first time, rapid particle melting dynamics, devolatilization and secondary fragmentation processes that influence the final characteristics of ash particles in high-speed videos, and measured the chemical alteration of the glass exposed to ultra-rapid melting.

## Experimental Setup

**Figure 1:**
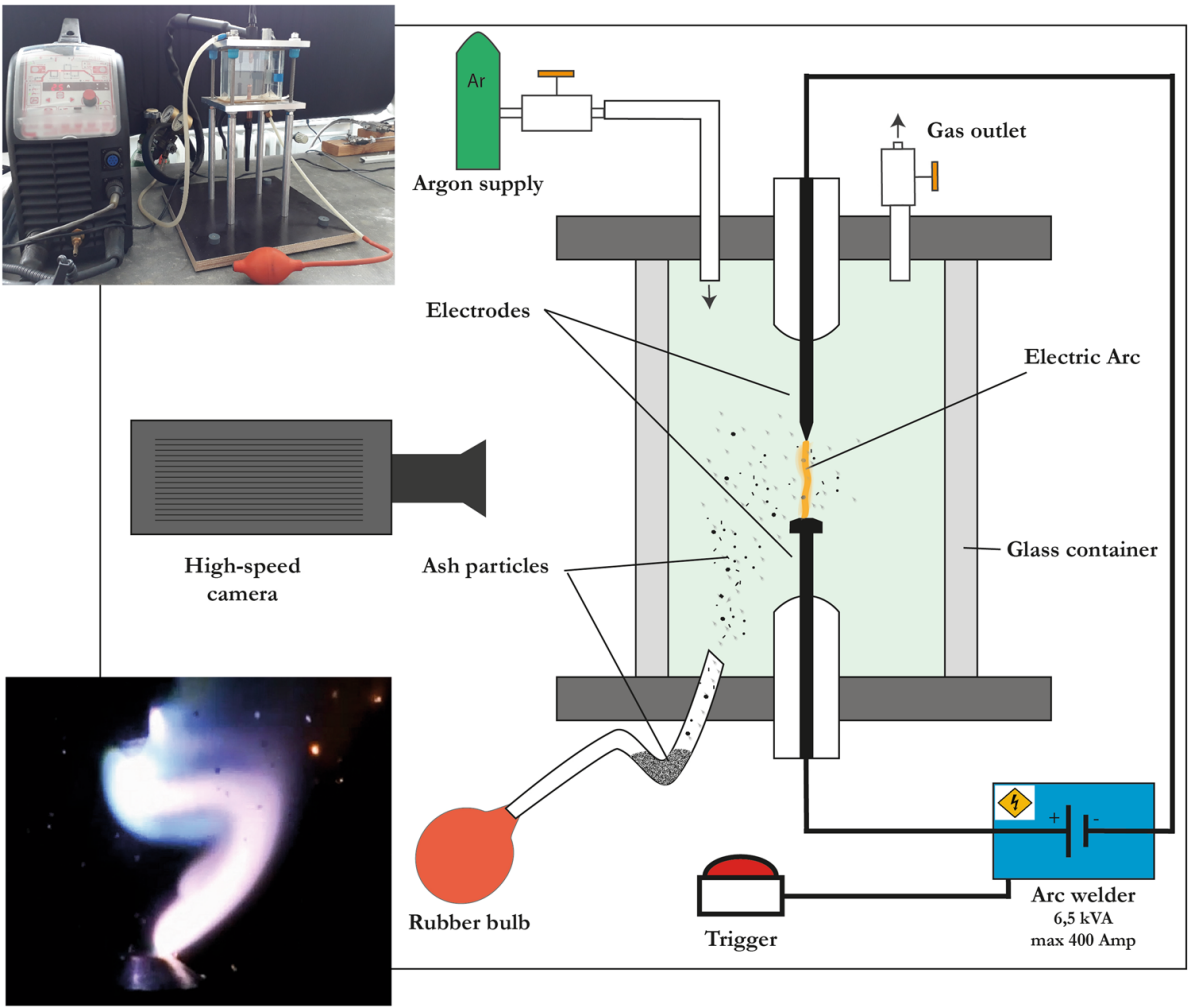
The experimental setup. An electric arc is created between two electrodes in an Argon-rich atmosphere. Subsequently, volcanic ash is injected into the cuboidal, 60 × 90 × 11.5 mm glass chamber, and can be recollected after the experiment. Upper inset shows a photograph of the device, the lower inset shows a still image of an actual experiment with glowing ash particles to the right; distance between the electrodes is approximately 1.3 cm.

In order to simulate the extreme temperature conditions of a lightning discharge in the laboratory, we have designed a novel experimental device (Fig. 1). In this setup, an electrical arc is created between two electrodes, which in turn are centrally positioned in a 620 cm^3^ glass chamber that is flooded with argon gas. The electrical discharge is generated by a TIG (tungsten-inert gas) arc-welding machine (CITOTIG DC 400). The arc-welder is capable of delivering a working current of up to 400 A with a maximum absorbed power of 13.8 kVA, however most of our experiments were performed at 25 A, due to the limitations of the glass housing and its potential damage. In order to create a plasma arc (i.e., the lightning bolt), a high frequency generator first induces a 1–3 kV electric spark, which establishes a conductive path between the electrodes. The high-current plasma arc then follows this path, which is analogous to the high-intensity backstroke in meteoric cloud-to-ground lightning. The continuous DC arc is then kept at constant current conditions throughout the experiment. The maximum temperature in the central part of a 25 A arc is estimated to reach at least 9000 K^16^, but might be lowered due to arc deformation during the experiment. By comparison, natural lightning return strokes attain temperatures peaking at 28,000 K and decreasing to about 15,000 to 10,000 K after 0.1 ms^13^. This “heat channel” is stable for at least 2 ms, even though the impulse voltage rise and decay time are in the order of only 50 µs^13^. High-speed analysis of natural volcanic lightning revealed after-stroke glow times of up to 26 ms, interpreted as a signal of high (but unspecified) temperatures^4,5^.

We performed ash-melting experiments on natural and synthetic grains from Sakurajima volcano (Japan), Chaitén volcano (Chile) and Laacher See volcano (Germany). For more details on the samples and analysis, see the Appendix. Our experiments were run in in two different manners:

(A) - injection experiments in which the ash sample is blown into the chamber from below upon ignition of the lightning arc (Fig. 1; supplementary video 1). Here, 1–3 g of ash is filled in a bent plastic pipe and injected using an argon-filled rubber ball. We used this setup to simulate the effect of lightning strikes on air-suspended particles with short interaction times. We define two interaction time windows, namely the *exposure time*, and the *thermal dwell time*. In our experiments, the exposure time defines the residence time of a moving injected particle in the heat channel established by the arc. This is equivalent to the natural case of a particle exposed to a heat channel established by a natural return stroke. The thermal dwell time is defined as the total duration the particle itself is at high temperatures (see supplementary information). In our experiments, the dwell time is established for temperatures above 900 °C, which is the minimum temperature recognizable by incandescence in the high-speed videos, as calibrated by a laser infrared thermometer. Exposure and thermal dwell times were estimated from high-speed video data collected at 100,000 fps with a NAC HX-3 camera. We traced glowing particles through the arc to establish exposure times, whereas dwell times were determined as the sum total of time particles spent within and beyond the arc, while remaining incandesdecent. For most particles, exposure times vary around an average value of 2 ms (1.2–2.9 ms within 1*σ*), and the thermal dwell time about 3–12 ms (average 7.8 ms), comparable to the upper limit of the duration of current flow associated natural volcanic lightning strokes^5^, or the time scale of heat dissipation around the plasma channel^13^.

(B) – stationary experiments: A single ash or lapilli-sized grain is placed onto the lower electrode (anode) prior to the experiment and subsequently exposed to the plasma arc. This configuration allows us to test and visualize fundamental effects of very high temperature and long exposure times of about 1–3 s on single particles.

## Lighting-induced particle modifications

### Morphologies and textures

**Figure 2:**
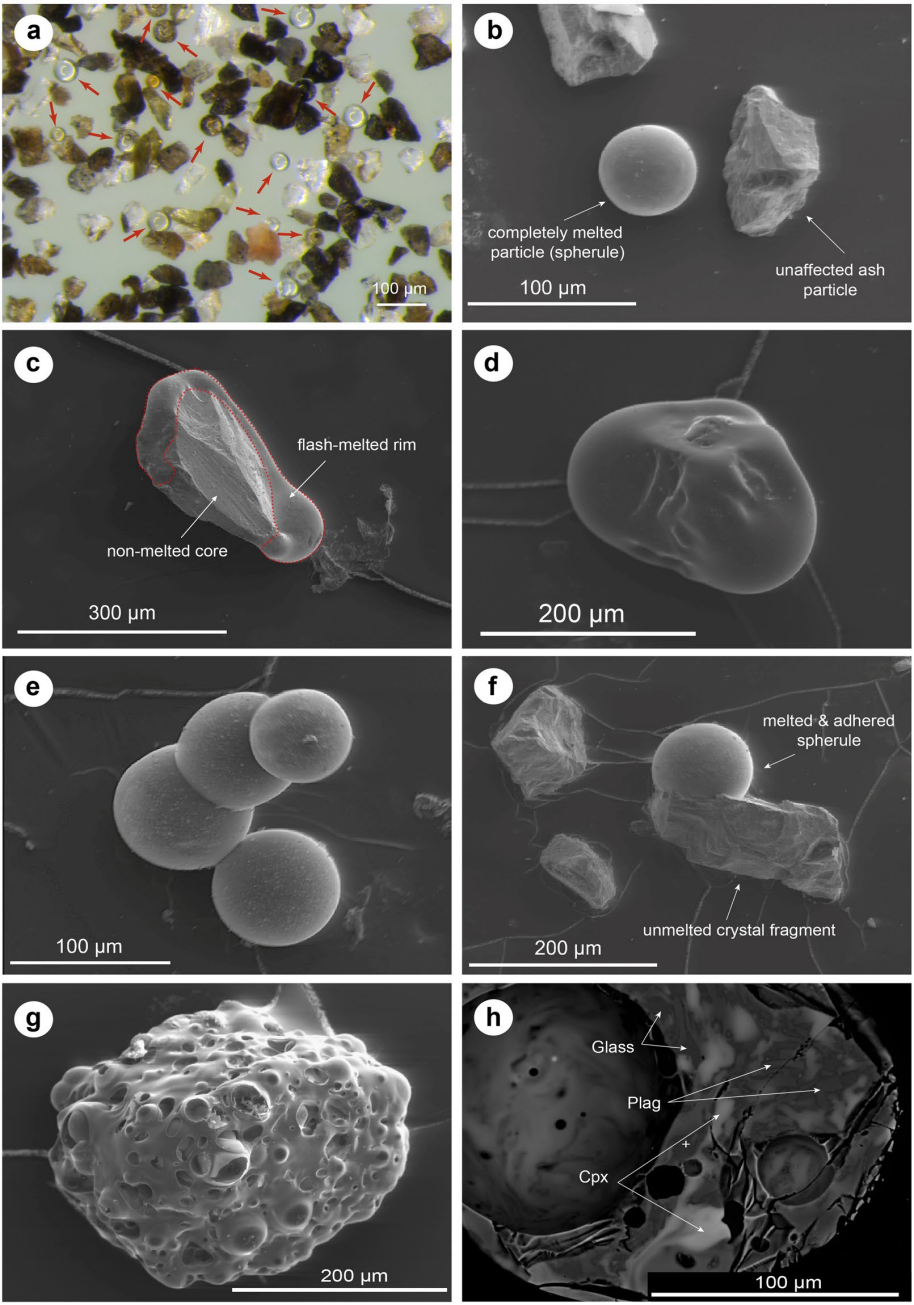
Morphology of experimentally produced lightning-affected particles (LAP) from natural ash (Sakurajima volcano, Japan). (**a**) Optical image of a sample with mostly spherical LAPs; (**b**) entirely re-melted, spherical particle (Type III), and pristine ash particles (Type I); **(c**,**d)** partially melted particles with a non-melted core; (**e**) agglomerate of spherical LAPs (Type IV); **(f)** spherical LAP attached to a pristine particle; (**g**) highly foamed obsidian chip (Chaitén); **(h)** BSE image of mingling structures of melted mineral phases and glass within a LAP. Note: vesicles formed during flash-melting.

We found that ash was affected by the electric arc in almost every injection-type experimental run with ash particles <250 µm (Fig. 2). The first-order process to occur was extremely rapid melting, herein referred to as *flash-melting*, of ash particles that interacted with the electric arc. For the sake of simplicity, we use the term *melting* for all cases of transformation from solid to a low viscosity liquid, irrespective the physical nature of the starting material, i.e. crystalline phase or glassy material. We anticipate that the resulting morphology of a lightning-affected particle (LAP) will, in general, depend on (a) the particle size, (b) the spatial and (c) the temporal relation of the particle-lightning interaction. The latter two are an expression of the path of the particle with respect to the hot central part of the plasma channel, i.e., the maximum temperature it experienced, and the dwell time above its 1-atm melting temperature, respectively. On the basis of our experiments, we classify the ash particles in 4 different categories, according to their degree of lightning interaction:

*Type I*. No visible interaction. Ash particles that are not affected by the electric arc, showing pristine morphologies, i.e., sharp edges and rough surface textures (Fig. 2b).

*Type II*. Partially melted particles. These are indicated either by smooth, liquid-like surface textures along the particle’s edges or to one side of the fragment (type IIa, Fig. 2c), or, for particles that may have undergone an higher degree of partial melting, comprising grains with only smooth surfaces, and no edges but non-spherical, irregular morphologies (type IIb, Fig. 2d). Sizes of the partially melted particles are commonly on the same order as the parent size fraction. We interpret the partial melting of particles as indication for an interaction along the (cooler) edge of the plasma channel^15^, and/or thermal dwell times that were too short to induce melting throughout the whole grain, resulting into surficial melting only. As demonstrated by Wadsworth *et al*.^17^ the latter effect strongly depends on the size of the particle. A further cause of incomplete melting could be that some particles are composed of different (mineral or glass) phases with different melting temperatures.

*Type III*. Completely melted particles (“spherules”). These have spherical forms that evidence temperatures well above the 1-atm liquidus of the bulk ash. The viscosity of these melts must have been low enough to allow re-rounding of the molten fragments into perfect spheres under the influence of surface tension (Fig. 2b), especially given the relatively short duration of the interaction (few ms). These LAPs often contain bubbles, suggesting element volatilization from the liquid (see, e.g., Figs 2h or 4a). In samples with relatively high water content (e.g., Chaitén obsidian), melting sometimes leads to the formation of extremely vesicular pumice fragments (Fig. 2g). In stationary particle experiments bubble formation was even evident in the nominally anhydrous phonolite glasses, suggesting vaporization of non-volatile silicate melt components (Fig. 4a, inset; supplementary video 6). According to our experiments there appears to be a particle size upper limit of ~250 µm for the formation of spheres, which is in accordance with theoretical studies^17^. Fluidal mingling structures of different mineral and glass phases can be observed in polished sections of spherical LAPs (Fig. 2h), reinforcing the notion that extremely low viscosities were attained during a short period of melting.

*Type IV*. Aggregated particles (Fig. 2e,f). These are clusters of up to 4 amalgamated spherical LAPs, as well as aggregates of both spherical and unmelted, pristine ash particles.

### Physical processes

**Figure 3:**
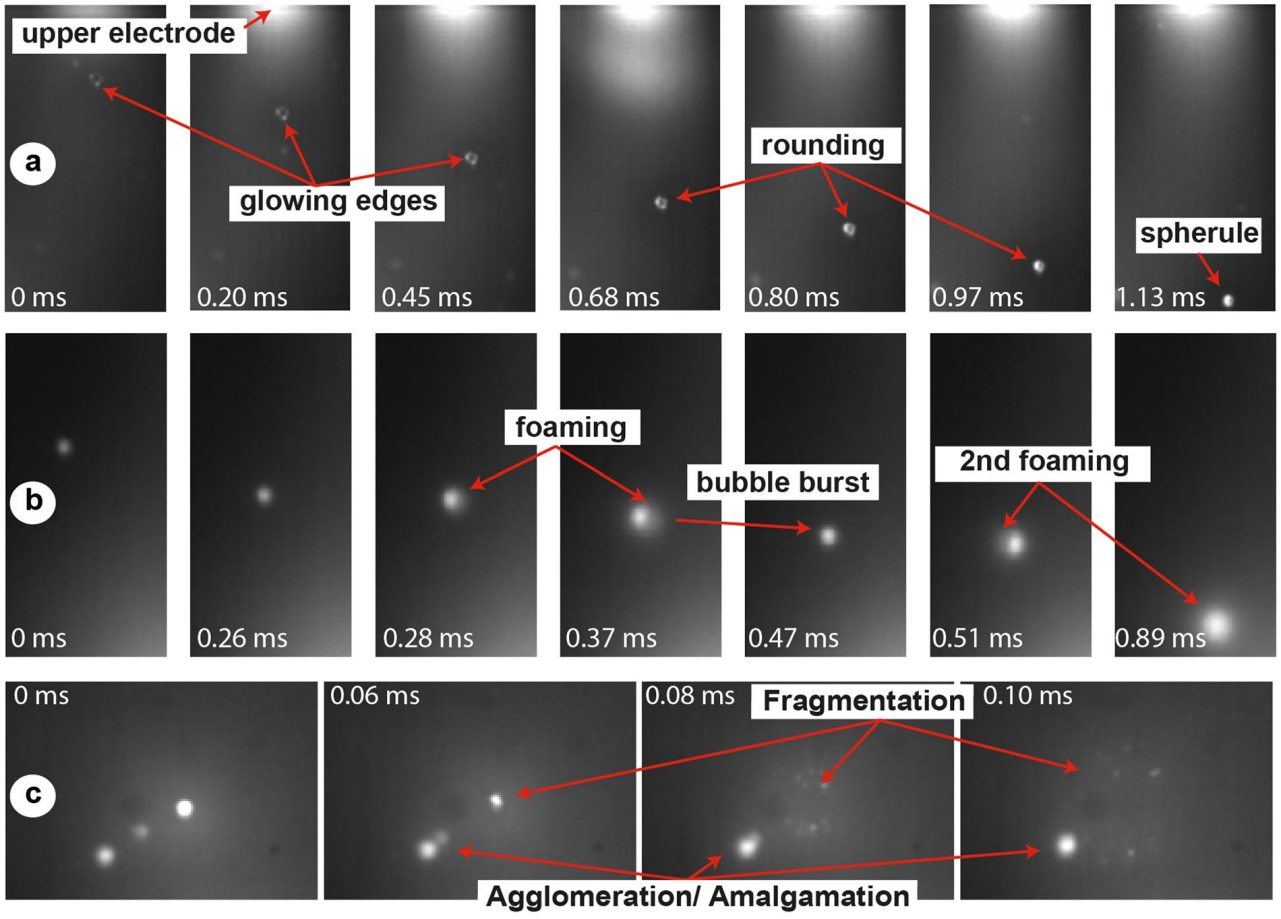
Still images of 100,000 fps high-speed videos of particle-lightning interactions. (**a**) Progressive heating and rounding of a ~200 µm sized Sakurajima ash particle to form a spherule (see Supplementary video 2). (**b**) A succession of inflation (foaming) and deflation (bubble burst) events of a water-bearing Chaiten obsidian fragment (initial size ~100 µm, see Supplementary video 3). (**c**) Full disintegration of a glowing Sakurajima particle into smaller fragments, and almost simultaneously a collision and agglomeration of two melted particles (see Supplementary videos 4 and 5).

100,000 fps high-speed videos of injection experiments revealed a variety of dynamic processes occurring during short ash-arc interactions that otherwise would not be observable from the static final forms of ash grains alone:

*Rounding*: As particles approach the hot zone of the arc, they start to glow, melt and transform onto increasingly spherical shapes (Figs 3a, 2b). Depending on particle size, spherule formation takes between several tens of µs to 2 ms. On larger fragments >100 µm, we often observe that melting initiates on exposed particle edges. When quenched prematurely, the partially melted particle morphologies are frozen in to form type II (*partially melted particles*) products (Fig. 2c,d; supplementary video 2).

*Vesiculation and foaming*: In various instances glowing particles can be observed to inflate rapidly during interaction with the arc, sometimes in several pulses with intermittent bubble rupture and deflation events (Fig. 3b; supplementary video 3). The liberation of a gas phase during this process often leads to an enhanced incandescence around the ash particle. The process of single bubble inflation can take as little as 20–30 µs (2–3 video frames). Vesiculation and inflation processes are particularly abundant in water-bearing obsidian samples and can lead to extremely pumiceous morphologies (Fig. 2g), but do also occur in anhydrous phonolite glass, suggesting that compounds and elements (e.g., Na) other than H_2_O contribute to particle outgassing.

*Fragmentation*: Rapid inflation due to bubble growth sometimes leads to explosive disintegration of a glowing ash particle into much smaller fragments (Fig. 3c; supplementary video 4).

*Collision and agglomeration*: If two or more melted particles collide, they amalgamate to form multi-spherule aggregates (Figs 3c and 2e,f; supplementary video 5).

*Complete vaporization*: High-speed video analysis suggests that particles in the size ranges investigated in our experiments (36–300 µm) do *not* entirely vaporize even when exposed to the highest arc temperatures (~9000 K) for the maximum dwell time (~12 ms). As the plasma temperatures in a lightning exceed, however, the vaporization temperature of every single constituent of a silicate magma, we postulate that a critical size limit exists below which particles will entirely vaporize in a natural lightning. Higher plasma temperatures (e.g. closer to channel axis^15^) will increase this size limit.

### Geochemical fingerprints of lightning-particle interaction

**Figure 4:**
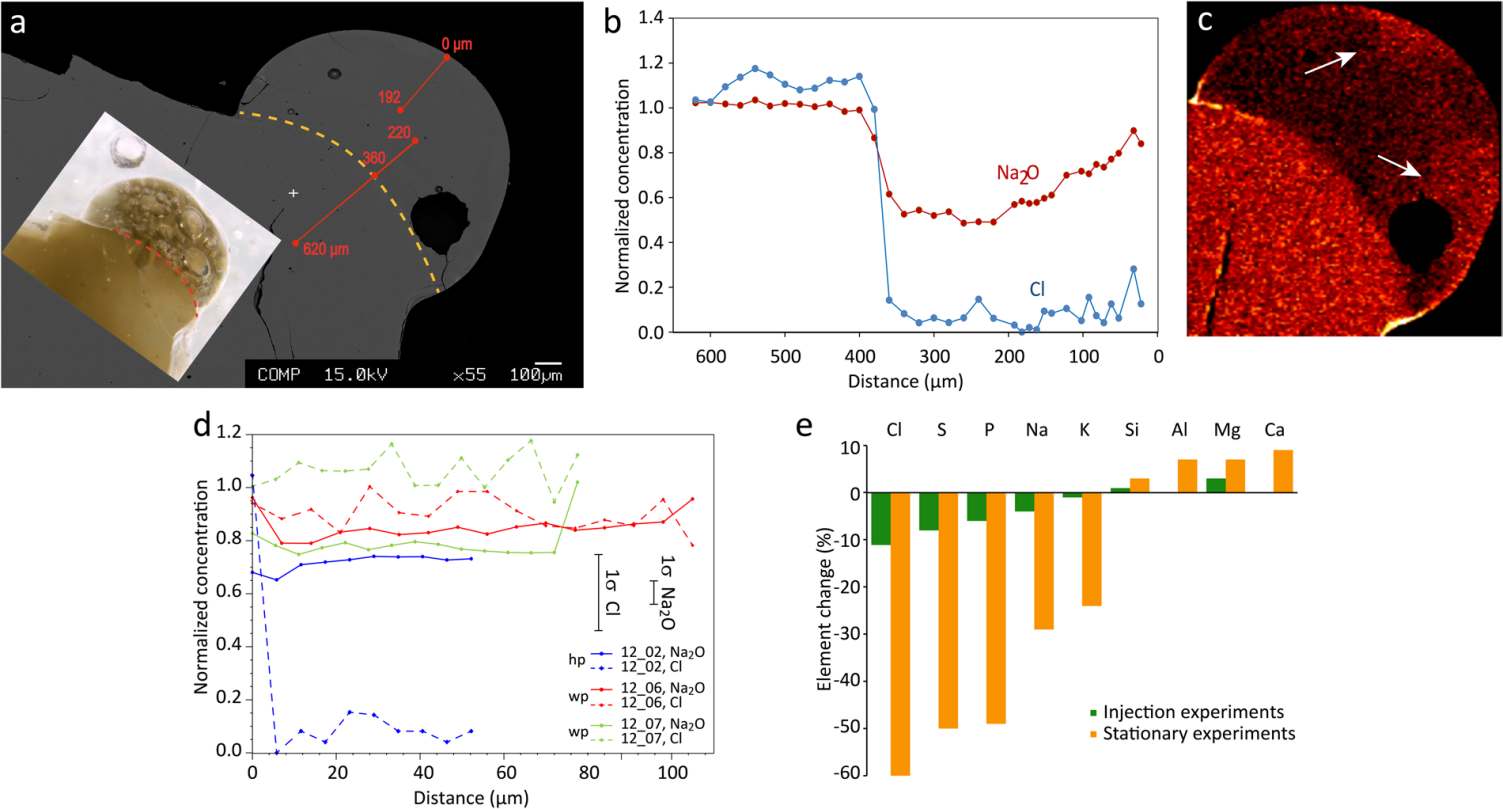
(**a**) Particle from a stationary experiment. Interaction with plasma arc led to melting and rounding of the upper edge, separated by the dashed yellow line from the less affected core. Inlay shows photomicrograph of the same particle with abundant bubbles formed as the droplet boiled. (**b**) Concentration profile from the core into the droplet showing a concentration gradient indicative of diffusion of Na. (**c**) EDS element map showing the distribution of Na in the host grain and droplet; light colors indicate high abundance. Note the Na-rich region in the droplet (arrows), which we interpret as a result of convection due to vesiculation and bubble rise. (**d**) Concentration profiles through LLST samples flash-melted during injection experiments (type III morphologies). “wp” and “hp” are profiles through the whole and half particle, respectively. (**e**) Order of thermal volatility as change in element concentration due to flash-melting. Note that the increase of Si, Al, Mg and Ca is a relative effect due to the loss of volatile elements. To mitigate strong bias introduced by individual concentration values below the detection limit, these were artificially assigned the value of the detection limit.

A total of 90 individual homogenized Lower Laacher See Tephra (LLST) phonolite glass grains (see Appendix) were analyzed for chemical alteration induced by the arc, of which 83 derived from injection experiments (partially and completely melted particle samples), and 7 from stationary particle experiments. The key chemical fingerprint of the arc stroke and the resultant flash-melting is the loss of Na, Cl, S and F: Concentrations of these elements show conspicuous depletions in the melted domains in injected and even more so in stationary particle experiments (Fig. 4). We interpret this as thermal volatilization due to the extreme heat experienced by the melt droplet. Given the increased exposure times of >1 s, stationary experiments are not a direct equivalent of the natural ash-lightning interaction, but help to confirm and better understand the chemical processes as they show the same, but amplified trends as injections experiments (e.g., Fig. 4e). The following order of thermal volatility, expressed through the degree of element depletion compared to the initial composition, and a proxy for the tendency of a particular element to escape the melt phase due to heating, can be constrained for our experiments: F > Cl > S ~ P > Na (Fig. 4e). None of the grains show spatially systematic concentration decreases or profiles, as would be expected for diffusive element transport from the melt’s interior toward the surface as a consequence of thermal element loss from the outer shell of a liquefied particle (Fig. 4d). Volatile element concentrations are generally inhomogenous throughout the whole grain, with very little to no directional trends. We attribute the lack of a systematic variation of the volatilized element depletion to the combined effects of diffusive and forced convective mass transport, in the sense that vesiculation and bubble motion may have influenced the final distribution of element across the grains (see supplementary video 6 of a vigorously boiling particle). Within the partially melted type II particles, the transition between un-melted parts and strongly melted regions that are affected by thermal loss of volatiles is demarcated by s-shaped element concentration profiles (Fig. 4b) typical for the juxtaposition of two melt batches with distinct compositional differences^18^.

Based on this chemical evidence, we suggest that the observed concentration and distribution of volatile elements can be readily explained by the combination of (1) thermal volatilization, (2) convective transport over distances of several tens of µm induced by moving vapor bubbles and (3) chemical diffusion processes acting over length scales defined by sharp concentration gradients. As volatiles are continuously lost from the outer liquid shell, convection is the first order process governing the distribution of volatile elements by mingling the depleted outer melt shell with undepleted subjacent material from the interior of the melt droplet, while diffusion is only locally efficient at flattening chemical concentration gradients between mingled fluid batches. Indeed, mingling is visually evident in BSE images of melted multiphase grains from natural ash, showing schlieren-like distribution of the different melt phases (Fig. 2h). A detailed analysis of element vaporization mechanisms is beyond the scope of this paper, but we briefly outline those for sodium and chlorine loss following published literature and simple chemical considerations.

Thermal volatilization of *sodium* from silicate melt droplets has been investigated in various experimental studies^19,20^ and is thought to be an important process during the formation of chondrules and tektites^20–23^. It may be described by the simplified first order reaction^19^

1 $$NaSi{O}_{2.5(l)}\rightleftharpoons N{a}_{(g)}+\frac{1}{2}{O}_{(g)}+Si{O}_{2(l)},$$

where NaSiO_2.5_ denotes a Na cation coupled to a tetrahedral silicate network unit (e.g., a Q^3^-species) and *SiO*_*2*_ a polymerized network unit (e.g., a Q^4^-species). The rate of the Na-loss reaction is negatively related to the oxygen fugacity of the surrounding atmosphere, with reducing conditions resulting in a rate increase^22^. *Chlorine* is thought to dissolve in silicate melts via Na- and Ca-complexes rather than bonding directly to the tetrahedral units^24,25^. A simplified loss reaction may thus be summarized by the third order reaction

2 $$\frac{1}{n}{M}^{n+}C{l}_{n(l)}+Si{O}_{2(l)}+\frac{1}{2}{O}_{(g)}\rightleftharpoons C{l}_{(g)}+{M}_{1/n}Si{O}_{2.5(l)},$$

with *M* being an alkali or alkaline earth cation and *n* its formal charge value. In contrast to Na in reaction (1), Cl needs to be oxidized in order to be liberated according to reaction (2).

In summary, volcanic lightning-ash interaction can thermally liberate volatile elements such as Cl, but also elements that are commonly not lost during volcanic degassing processes, e.g., Na. Available experimental data and general chemical considerations suggest that the induced chemical modifications are sensitive to oxygen fugacity of the surrounding atmosphere, which is likely to vary by several orders of magnitude within volcanic plumes.

## Estimates on particle temperature during flash-melting

The temperature evolution of LAPs is largely unknown. First theoretical considerations were given by Wadsworth *et al*.^17^, assuming a lightning temperature of 3273 K and conductive as well as convective heat flow within the particles. Here, we provide constraints on the thermal history of LAP by modelling the average temperature achieved by individual ejected particles in our experiments on the basis of the quantified degree of Na volatilization, i.e. measured concentration normalized to initial concentration, *c*_*Na*_/*c*_0*,Na*_. This was done by assuming an Arrhenian temperature dependence of the reaction coefficient $k(T)={k}_{0}\times \exp (\frac{-{E}_{a}}{RT})$ of reaction (1), the relation $k=-\mathrm{ln}(\frac{{c}_{Na}}{{c}_{0,Na}})\times \frac{D}{6t}$ (see Supplementary Figures 1, 2, and 3) and measured *c*_*Na*_/*c*_0*,Na*_ ratios (where *k*_*0*_ is the prefactor, *E*_*a*_ the activation energy, *R* the universal gas constant, *D* the particle diameter and *t* the time). By combining and regressing experimental data from Tsuchiyama *et al*.^22^ and Yu & Hewing^20^, we derive a generalized *k*_*0*_ as a function of oxygen fugacity and composition (*X*). Composition is expressed in terms of the atomic ratio of Mg + Fe + Ca to Si + Na. This results in the following general approximation for *k* of reaction (1):

3 $$\mathrm{ln}\,k(T,f{O}_{2},X)=(7.334+3.08\times \frac{Mg+Fe+Ca}{Si+Na}-0.265\times \,\mathrm{ln}\,f{O}_{2})-\frac{467}{RT}.$$

The precise oxygen fugacity within the experimental chamber is not known, but we conservatively assume around 1% residual O_2_ inside the glass container (using 0.1% O_2_ as a minimum, the calculated temperatures decreases by about 3%). The overall thermal dwell time for most particles is 3–12 ms. Assuming that completely melted LAP of type III showing considerable Na depletion, i.e., *c*_*Na*_/*c*_*0,Na*_ < 0.9 were indeed exposed up to 10 ms, we calculate temperature between 3290 K and 3490 K for particles between 70–130 µm. At such high temperatures the melt viscosities are approximately 10^−1.3^ to 10^−1.5^ Pas^26^ with corresponding structural relaxation times of 10^−11.3^ to 10^−11.5^ s, derived from the Maxwell relation and an elastic shear modulus G_∞_ of 10^10^ Pa^27^. These low viscosity conditions are the primary reason for such short time scales associated with the rounding and vesiculation. Our experimental conditions agree reasonably well with the lower end of temperatures expected in natural lighting plasma channels^13^ and with natural thermal dwell time of the ash. Thus, the melt temperatures estimated from our experiments can provide a basis to interpret and estimate the thermal histories of natural LAPs, with the caveat that experiments capture just a segment of natural lightning’s energy spectrum.

## Conclusions

- Our arc-welder based experimental setup can simulate lightning-related rapid heating events of volcanic ash and produces a range of modified ash products whose physical and chemical characteristics depend on the temperature and style of exposure of ash grains to the electric discharge. Future design modifications could enable investigations on the effects of oxygen fugacity on element volatilization, as this is expected to vary widely in natural volcanic plumes.
- Experimentally produced spherical ash particle morphologies and sphere aggregates resemble those described from natural ash deposits. These forms, along with dynamic evidence for particle melting and rounding captured in high speed videos, constitute strong evidence that such features originate from volcanic lightning, which is consistent with earlier interpretations. We have also generated a range of previously unrecognized morphologies, including partly molten and vesicular clasts.
- The high temperatures achieved during ash-plasma arc interaction initiates a range of physical and chemical processes that occur on the milli- to microsecond timescales. In addition to rounding of the melt droplet due to surface tension – and vesiculation and fragmentation in the case of water-rich ash- chemical mass transport and element loss within the liquefied droplets is important, driven by (forced) convection stemming from bubble motion.
- Maximum melt temperatures for ash in the range 70–130 µm were modeled based on the degree of thermal sodium loss and range between 3290 K and 3490 K. These temperature estimates may capture some of natural lightning’s energy characteristics, which could lead to better constraints on the thermal history of particles following the exposure to volcanic lightning.
- Thermal volatilization of halogens, sulfur, phosphorus and sodium was observed in our experiments. Some of these elements, in turn, have the capability to affect atmospheric chemistry (e.g., ozone destruction) and aerosol formation. We expect that in natural lightning-affected plumes, the degree of element loss will differ depending on grain size distribution and elemental concentration in the ash. Future studies should focus on chemical processing of natural plumes, in which the variability in the ambient oxygen fugacity is expected to play an additional role.

### Appendix: Sample material and analysis

We used three types of natural and synthetic ash samples for our experiments: (a) Natural ash from Sakurajima volcano, Japan, collected on Oct 30 2013 during deposition from the plume near Kurokami observatory, located 3.5 km east of the then-active Showa crater. These andesitic samples consist of both glassy and poly-crystalline components (Plag + Cpx + Opx ± Ol), as well as single crystal fragments. The ash was sieved into six different size fractions between 36 µm and 300 µm. (b) Obsidian fragments from the 2008 eruption of Chaitén volcano, Chile. This aphyric, hydrous rhyolitic glass (1.4 to 1.7 wt.% H_2_O) was explosively erupted as obsidian bombs in the first two weeks of activity^28^. Parts of the samples were crushed and sieved into the size fractions 36–100 µm, 100–150 µm, >150 µm. (c) Phonolitic glass synthesized from pumice clasts from the Lower Laacher See Tephra (LLST), Eifel, Germany. Samples were finely ground and remelted twice at 1300 °C in a vertical tube furnace at one-atmosphere and uncontrolled oxygen fugacity conditions, to obtain a chemically homogenous, dry, alkali-rich glass. The glass was coarsely crushed and sieved into six different size fractions between 36 µm and 300 µm.

High-speed videos of experiments were made using a NAC HX-3 high-speed camera with a Zeiss 100 mm macro lens, at a frame rate of 100,000 fps and a shutter speed of 1/100,000 s, and a Edgertronic SC2+ high-speed camera at 4000 fps. Pre- and post-experimental particles were analyzed for morphological alterations using a 120× optical stereomicroscope, and a Zeiss DSM 942 scanning electron microscope operating in secondary electron imaging mode. Finally, bulk major element chemistry and variations thereof were determined by electron probe microanalysis using a JEOL JXA-8200 at the Institute for Geosciences in Mainz. For more details on measurement conditions and geochemical data see Supplementary Information 1 and 2.

## Electronic supplementary material

Video 1 - Lightning Experiment

Video 2 - Particle Rounding

Video 3 - Particle Foaming

Video 4 - Particle Fragmentation

Video 5 - Particle Aggregation

Video 6 - Stationary Boiling

Supplementary Information

Dataset 1

## References

[1] Mather TA, Harrison RG (2006). Electrification of volcanic plumes. Surv. Geophys..

[2] James MR (2008). Electrical charging of volcanic plumes. Space Sci. Rev..

[3] van Eaton AR (2016). Volcanic lightning and plume behavior reveal evolving hazards during the April 2015 eruption of Calbuco volcano, Chile. Geophys. Res. Lett..

[4] Cimarelli C (2016). Multiparametric observation of volcanic lightning: Sakurajima Volcano, Japan. Geophys. Res. Lett..

[5] Aizawa K (2016). Physical properties of volcanic lightning: Constraints from magnetotelluric and video observations at Sakurajima volcano, Japan. Earth Planet. Sci. Lett..

[6] McNutt SR, Davis CM (2000). Lightning associated with the 1992 eruptions of Crater Peak, Mount Spurr Volcano, Alaska. J. Volcanol. Geotherm. Res..

[7] Hoblitt RP (1994). An experiment to detect and locate lightning associated with eruptions of Redoubt Volcano. J. Volcanol. Geotherm. Res..

[8] McNutt SR, Williams ER (2010). Volcanic lightning: global observations and constraints on source mechanisms. Bull. Volcanol..

[9] Behnke SA, McNutt SR (2014). Using lightning observations as a volcanic eruption monitoring tool. Bull. Volcanol..

[10] Behnke SA, Bruning EC (2015). Changes to the turbulent kinematics of a volcanic plume inferred from lightning data. Geophys. Res. Lett..

[11] Lane SJ, Gilbert JS (1992). Electric potential gradient changes during explosive activity at Sakurajima volcano, Japan. Bull. Volcanol..

[12] Cimarelli C, Alatorre-Ibargüengoitia MA, Kueppers U, Scheu B, Dingwell DB (2014). Experimental generation of volcanic lightning. Geology.

[13] Paxton AH, Gardener RL, Baker L (1986). Lightning return stroke. A numerical calculation of optical radiation. Phys. Fluids.

[14] Genareau K, Wardman JB, Wilson TM, McNutt SR, Izbekov P (2015). Lightning-induced volcanic spherules. Geology.

[15] Gennareau K, Gharghabi P, Gafford J, Mazzola M (2017). The Elusive Evidence of Volcanic Lightning. Scientific Reports.

[16] Bott JF (1966). Spectroscopic measurements of temperatures in an argon plasma arc. Phys. Fluids.

[17] Wadsworth FB (2017). Size limits for rounding of volcanic ash particles heated by lightning. J. Geophys. Res.-Sol. Ea..

[18] Zhang Y (2010). Diffusion in Minerals and Melts: Theoretical Background. Rev. Mineral. Geochem..

[19] Corrigan G, Gibb FG (1979). The loss of Fe and Na from a basaltic melt during experiments using the wire-loop method. Mineral. Mag..

[20] Yu Y, Hewins RH (1998). Transient heating and chondrule formation: Evidence from sodium loss in flash heating simulation experiments. Geochim. Cosmochim. Ac..

[21] Taylor SR (1961). Distillation of alkali elements during formation of Australite flanges. Nature.

[22] Tsuchiyama A, Nagahara H, Kushiro I (1981). Volatilization of sodium from silicate melt spheres and its application to the formation of chondrules. Geochim. Cosmochim. Ac..

[23] Folco L, Glass B, D’Orazio M, Rochette P (2010). A common volatilization trend in Transantarctic Mountain and Australasian microtektites: Implications for their formation model and parent crater location. Earth Planet. Sci. Lett..

[24] Stebbins JF, Du L-S (2002). Chloride ion sites in silicate and aluminosilicate glasses: A preliminary study by 35Cl solid-state NMR. Am. Mineral..

[25] Sandland TO, Du L-S, Stebbins JF, Webster JD (2004). Structure of Cl-containing silicate and aluminosilicate glasses: A 35Cl MAS-NMR study. Geochim. Cosmochim. Ac..

[26] Giordano D, Russel JK, Dingwell DB (2008). Viscosity of magmatic liquids: A model. Earth Planet. Sci. Lett..

[27] Webb SL, Dingwell DB (1995). Viscoelasticity. Rev. Mineral. Geochem..

[28] Castro JM (2012). The role of melt-fracture degassing in defusing explosive rhyolite eruptions at volcán Cahitén. Earth Planet. Sci. Lett..

