# Supplementary material for: First experimental observations on melting and chemical modification of volcanic ash during lightning interaction
Source: Sci Rep. 2018 Jan 23;8:1389. doi: 10.1038/s41598-018-19608-3 (PMC5780474; doi:10.1038/s41598-018-19608-3)
Supplement: Supplementary file 7 — Supplementary Information [file 41598_2018_19608_MOESM7_ESM.pdf]

Supplementary Information:

First experimental observations on melting and chemical modification of volcanic ash during lightning interaction

S.P. Mueller[1], C. Helo[1], F. Keller[1], J. Taddeucci[2], J.M. Castro[1]

[1]Intitute of Geosciences, University of Mainz, J.-J.-Becherweg 21, D-55122 Mainz, Germany

[2]Istituto Nazionale di Geofisica e Vulcanologia, Via di Vigna Murata 605, 00143, Rome, Italy

EPMA measurement conditions

Analytical conditions were 15 kV acceleration voltage and 12 nA beam current. A defocused beam of 5 to 10 µm was used. Sodium loss was found to be negligible for the measurement time used. Major elements were calibrated against VG-A99 (SiO_2, FeO), VG-2 (Al_2O_3, CaO, MgO), orthoclase (K_2O), tugtupite (Na_2O, Cl) and other mineral standards. The Smithsonian standard "Obsidian" was interspersely analyzed to check for signal drift and quality of the measurements. 1σ error (n= 25) for SiO_2 and Na_2O was 0.4 % and 2.0 %, respectively. See suppl. data Table for error of other elements.

Reaction kinetics as function of particle size, oxygen fugacity and composition

For the first order reaction (1), the conversion rate $\dot{\xi} = \frac{\mathrm{d}Na}{\mathrm{d}t}$ describes the change in the amount of sodium with time. Since the reaction is heterogeneous, occurring at the melt-air interface, $\dot{\xi}$ depends on the concentration of sodium c_{Na} and on the surface A of the melt droplet and the rate of reaction υ can be defined as $= \frac{\dot{\xi}}{A}$. The standard solution for the reaction rate $k = \frac{\upsilon}{c_{Na}}$ coefficient becomes $-\ln\left(\frac{c_{Na}}{c_{0,Na}}\right) = kt\frac{6}{D}$,where D is the diameter of the melt droplet (cf. Tsuchiyama et al. 1981). The Arrhenian relation $\ln(k) = \ln(k_0) + \frac{E_a}{RT}$ describes the temperature dependence of the reaction rate coefficient (suppl. fig. 1). Regressing data by Tsuchiyama et al. 1981 that describe the rate of sodium loss of small melt droplets as a function of temperature and oxygen fugacity, gives an activation Energy E_a of 467 kJ mol^{-1}, and the following dependence of the prefactor k_0 on the partial pressure of oxygen P_{O2}:

$\ln(k_0) = -21.664 - 0.2646 \times \ln(P_{O_2})$ (suppl. fig. 2). This agrees with the approximation given in the original publication of k_0 proportional to $P_{O2}^{-1/4}$. We also describe the influence of melt composition on kinetics of reaction (1) in terms of the prefactor. The data by Yu and Hewins (1998)

were therefore regressed using the cation ratio (Mg+Fe+Ca)/(Si+Na) to describe the chemical composition (Suppl. Fig. 3). Implementing both prefactor dependencies into the Arrhenian relation provides an approximation of the reaction kinetics of (1) accounting for composition and oxygen fugacity:

$$\ln k(T, fO_2, X) = \left(7.334 + 3.08 \times \frac{Mg+Fe+Ca}{Si+Na} - 0.265 \times \ln fO_2\right) - \frac{467}{RT}.$$

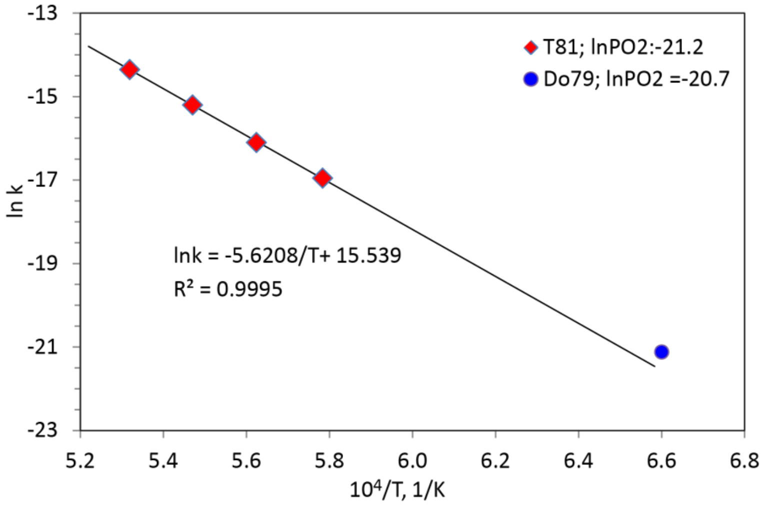

Supplementary Figure 1 *Arrhenian plot with regressed data Tsuchiyama et al. (1981). Data point "Do79" (Donaldson 1979) is not used in the regression.*

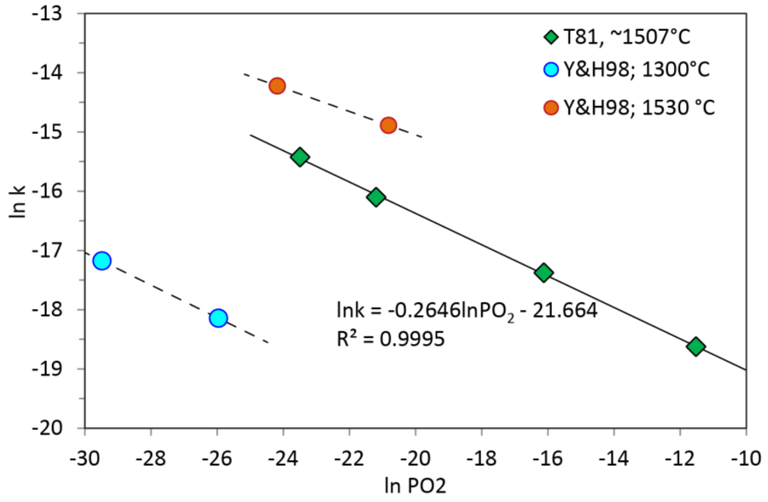

Supplementary Figure 2 *Dependence of the sodium loss reaction kinetics on the partial pressure of oxygen. Same data set as Suppl. Fig. 1. Data by Yu and Hewins (1998) agree well with the slope of the regression, but are shifted along the ordinate due to differences in temperature and composition.*

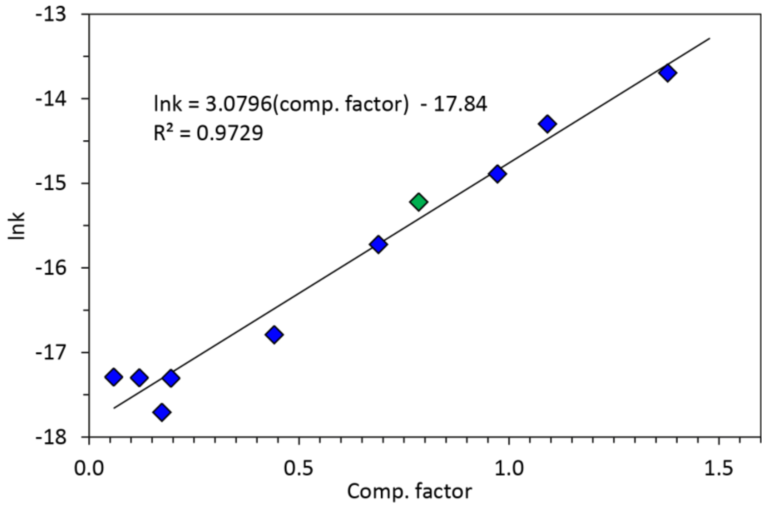

Supplementary Figure 3 *Dependence of the sodium loss reaction kinetics on melt composition. Data is from Yu and Hewins (1998). Comp. factor is the cation ratio (Mg+Fe+Ca)/(Si+Na).*

Estimation of arc dimensions, exposure and thermal dwell time

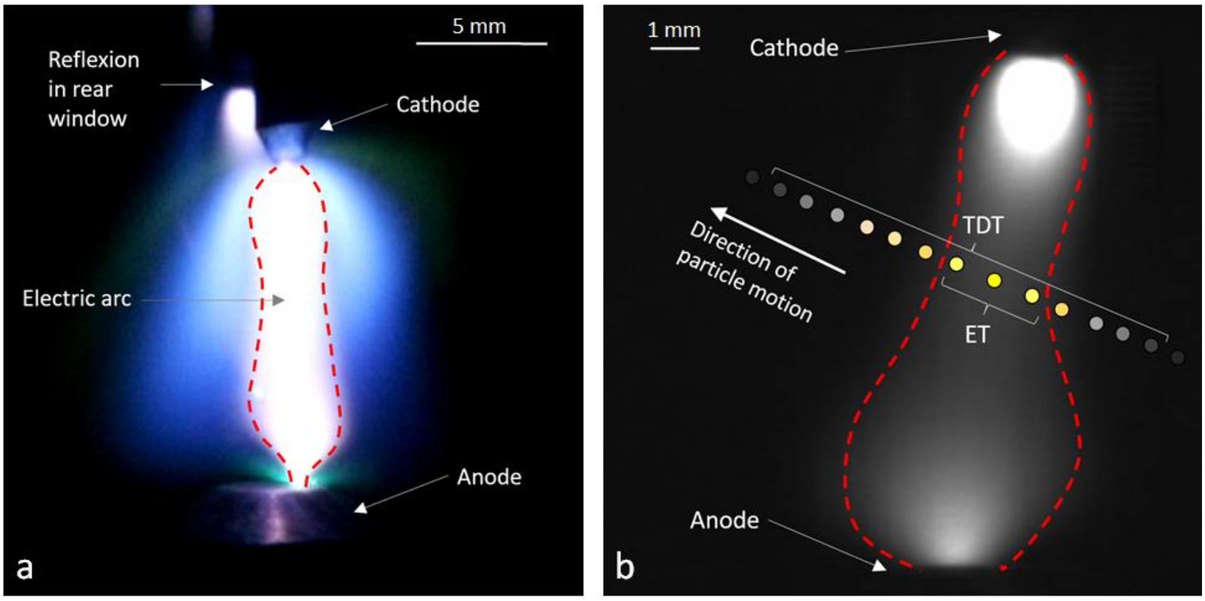

Supplementary Figure 4 *Dimensions of the electric arc (outlined with dashed line) in video still images from (a) a Edgertronic SC2+ camera at 4000 fps, and (b) a NAC HX-3 camera at 100,000 fps. In (b), the exposure time (ET) and thermal dwell time (TDT) are schematically depicted: ET – residence time of the moving particle IN the electric arc. TDT: total duration the particle is at T>900°C, which is the minimum temperature recognizable by incandescence in the high-speed videos. The average ET value measured is 2.1 ms (σ = 0.83, n= 22), the average TDT value is 7.8 ms (σ = 4.6, n= 23).*

Supplementary video captions

Supplementary video 1: 4000 fps high speed-video of an injection experiment. Particles are injected from the lower right corner into the established lightning arc at ~0:46s. Note the various degrees of incandescence of particles upon exposure to the arc. The sequence 1:24-1:33 nicely displays a rounding process, including signs of volatile liberation at the beginning (bubble growth and burst, expressed as pulsation) of a particle on the left edge of the arc. Natural duration of the video is 1.2s, the distance between the electrodes is 13 mm for scale.

Supplementary video 2: 100,000 fps video showing the rounding process of a ~200 µm sized Sakurajima ash particle, magnified in the right window. The melting process (indicated by incandescence) can be seen to start on the particles' edges. The process of sphere formation takes about 1.5 ms. Natural duration of the video sequence is 1.625 ms, the field of view (width) of the left window is 6.3 mm.

Supplementary video 3: 100,000 fps video showing two rapid (20-30 µs) inflation (bubble growth) events of a ~100 µm sized Chaiten obsidian particle at 00:02 and 00:04s, intermitted by a deflation, interpreted as burst of the first bubble. The right window shows a magnified view of the particle. Natural duration of the video sequence is 1.125 ms, the field of view (width) of the left window is 6.3 mm.

Supplementary video 4: 100,000 fps video showing a rapid disintegration (fragmentation) of a ~50 µm sized Sakurajima ash particle at 00:10s.The right window shows a magnified view of the particle. Natural duration of the video sequence is 1.333 ms, the field of view (width) of the left window is 6.3 mm.

Supplementary video 5: 100,000 fps video showing the amalgamation/agglomeration process of two ~30-50 µm sized Sakurajima ash particles at 00:01s, simultaneous to the fragmentation event of video 4. The right window shows a magnified view of the particles. Natural duration of the video sequence is 1.125 ms, the field of view (width) of the left window is 6.3 mm.

Supplementary video 6: 100,000 fps video of a ~2 mm sized particle of dry phonolitic glass (LLST), sitting on the lower electrode ('stationary experiment'). The particle shows, despite the anhydrous character of the melt, vigorous and highly dynamic degassing features (bubble nucleation, growth, coalescence, and rupture at the surface). The total natural duration of the video is 0.89 s, intermitted by a slowed-down, 2.88 ms long sequence to highlight bubble coalescence processes in the droplet. The field of view (width) is 6.3 mm.
